# Long-term clade-wide shifts in trilobite segment number and allocation during the Palaeozoic

**DOI:** 10.1098/rspb.2022.1765

**Published:** 2022-12-21

**Authors:** Melanie J. Hopkins, Rebecca To

**Affiliations:** ^1^ Division of Paleontology (Invertebrates), American Museum of Natural History, New York, NY 10024, USA; ^2^ Department of Ecology and Evolution, University of Michigan Ann Arbor, MI 48109, USA

**Keywords:** Arthropoda, constraint, evo-devo, evolutionary trends, segmentation, Trilobita

## Abstract

Arthropods are characterized by having an exoskeleton, paired jointed appendages and segmented body. The number and shape of those segments vary dramatically and unravelling the evolution of segmentation is fundamental to our understanding of arthropod diversification. Because trilobites added segments to the body post-hatching which were expressed and preserved in biomineralized exoskeletal sclerites, their fossil record provides an excellent system for understanding the early evolution of segmentation in arthropods. Over the last 200 years, palaeontologists have hypothesized trends in segment number and allocation in the trilobite body, but they have never been rigorously tested. We tabulated the number of segments in the post-cephalic body for over 1500 species, selected to maximize taxonomic, geographical and temporal representation. Analysis reveals long-term shifts in segment number and allocation over the 250-million-year evolutionary history of the clade. For most of the Palaeozoic, the median number of segments in the body did not change. Instead, the total range decreased over time and there was long-term increase in the proportion of segments allocated to the fused terminal sclerite relative to the articulated thoracic region. There was also increased conservation of thoracic segment number within families. Neither taxonomic turnover nor trends in functionally relevant defensive behaviour sufficiently explain these patterns.

## 1. Introduction

Arthropods are invertebrate animals with an exoskeleton, paired jointed appendages and a segmented body. The final segmental composition of the body in many arthropods is attained during post-embryonic development through a series of moults. Hemianamorphic arthropods are characterized by undergoing a phase of moulting (ecdysis) during which new segments are added to the body followed by a phase during which ecdysis and growth continues even though no new segments are added to the body. Arthropods with this mode of segmentation occur across the phylum, including some crustaceans, some myriapods and some extinct arthropods like trilobites [1]. Trilobites, in particular, have been useful for studying segmentation patterns because their exoskeleton was highly biomineralized from an early post-embryonic stage, and thus they have a rich fossil record that includes complete developmental series for many species [2].

The mature trilobite exoskeleton consisted of an anterior cephalic shield comprised of fused segments, and a trunk consisting of a thorax with narrow, similarly shaped articulated tergites^1^ and a posterior pygidial shield comprised of fused segments [3,4]. At regular to semi-regular intervals during ecdysis, segments were generated at the posterior part of the pygidium; some of these segments were ultimately released into the thorax from the anterior margin of the pygidium [5,6]. Thus segment identity in the trunk shifted during post-embryonic development, with all post-cephalic segments initially belonging to the pygidium, but the subset of earliest-generated segments eventually belonging to the thorax. The terminal number of thoracic tergites in many species was invariant, in some cases conservatively so across all members of higher level clades. In other species, the terminal number of thoracic tergites varied as much as 10% [7].

A 1920 compilation based on a small number of specimens reported a decrease in the average number of thoracic tergites from the earliest Cambrian and more protracted increase in the number of pygidial segments across the Palaeozoic [8]. Changes in the pygidial segment count along with an increase in the relative size of the pygidium [8,9] underlie the proposed trend towards increased ‘caudalization’ of the pygidium, an idea that also encompasses an apparent trend towards increased differentiation in the morphological expression of segments in the pygidium relative to the thoracic tergites [8,10,11]. Together these observations predict long-term trends in shifting patterns of segment allocation and expression in the mature trilobite body.

## 2. Materials and methods

We tabulated the number of segments in the trunk for 1589 trilobite species from images sourced from primary and secondary literature and museum catalogues (electronic supplementary material, Dataset S1). We limited our search to complete, well-preserved, articulated specimens, necessarily excluding the vast majority of named trilobite species (*N* > 22 000, [12]) for which the exoskeleton is known only from disarticulated sclerites. We also focused our search on specimens with stratigraphic age information precise enough to place within global geological series. Within these constraints, we endeavoured to maximize geographical, stratigraphic and taxonomic representation (electronic supplementary material, tables S1–S3 and figures S1 and S2), but recognize that there are potential local environmental controls on the occurrence of articulated specimens in the rock record ([13–15]; see electronic supplementary material for discussion). Images were sourced primarily from literature in English, but also from literature in Chinese, Czech, French, German, Japanese, Norwegian, Russian and Swedish (electronic supplementary material, Datasets S2 and S3).

**Figure 1.**
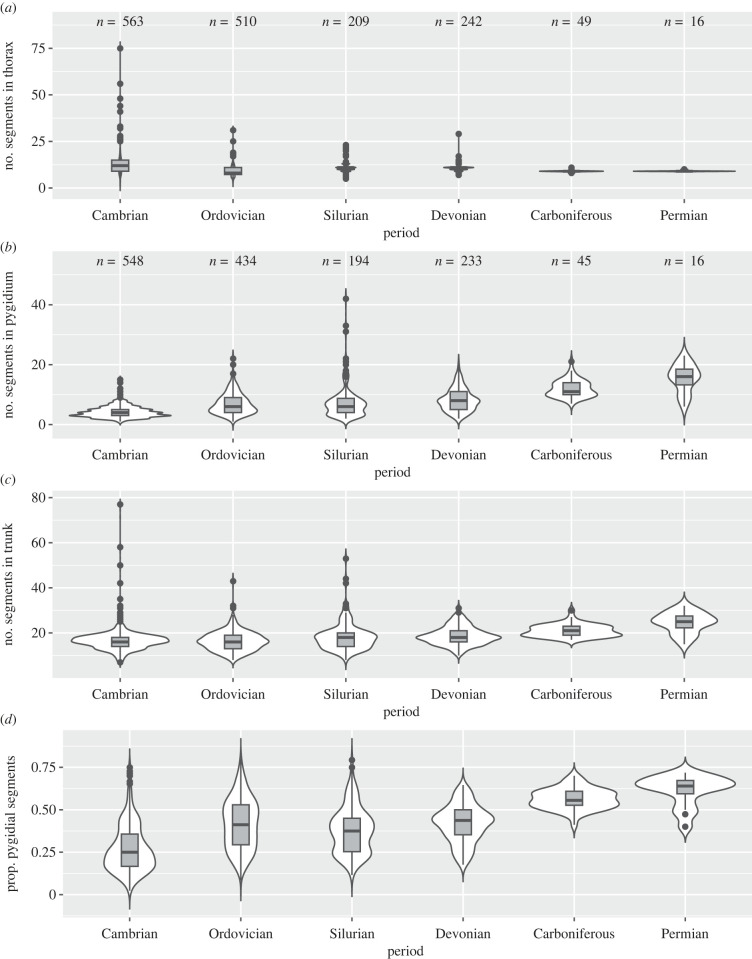
Number of segments in trilobites during each period of the Palaeozoic. Violin plots (white) and box plots (dark grey) show the distribution and median/quartiles, respectively, in segment count in the thorax (*a*), pygidium (*b*), entire trunk (*c*) and proportion of the trunk segments that are fused in the pygidium (*d*). Sample sizes for each period are shown at top of each panel; sample sizes in (*c*,*d*) are the same as (*b*). See table 1 for results of statistical analysis and electronic supplementary material, table S4 for median values. (Online version in colour.)

Because the distributions of segment numbers in different time intervals show skewed distributions with heavy tails and different sample sizes (electronic supplementary material, figures S3–S5), common approaches for testing for differences in means may be inappropriate. Instead we test for location shifts in the median among multiple groups using the single-step procedure of Richter & McCann [16]. The procedure does not assume normal distributions or equal samples sizes and provides strong control of familywise error rates. Although it does assume identical distributions among groups, the procedure is more robust to violations of this assumption than other tests for differences between medians/means, especially when the distributions with high skew and kurtosis also have the larger sample sizes [17], as is frequently the case here (electronic supplementary material, figures S3–S5). Richter & McCann [18] also proposed a sequential (step-down) permutation procedure, including an automatized but conservative realization of the procedure (also referred to as conservative maximal subsets). The step-down procedure is more powerful than the single-step approach but is also considerably more computationally demanding for any dataset with more than a few groups. We confirmed that the results for a smaller number of groups (all comparisons across periods, the basis of figure 1) were consistent between the single-step approach (table 1) and the conservative maximal subsets implementation of the step-down approach (electronic supplementary material, table S5). For all other analyses, we used the single-step approach. Because this is a permutation test, each new implementation returns a slightly different set of *p*-values, but not enough to change the interpreted significance of the results. The number of permutations for all applications was 10 000.

**Table 1.** Results of differences in median using single-step permutation procedure [16] at coarse timescale (geological periods). Values are *p*-values for the null hypothesis that the median number of segments in each comparison is not different; significant *p*-values are italicized. Results were obtained from single analysis and are organized separately only to make it easier to see both sequential comparisons and long-term trends. Figure 1 and electronic supplementary material, table S4 for median values.

coarse timescale (geological period)	thorax	pygidium	trunk	proportion
sequential				
Cambrian–Ordovician	*0.0026*	0.7291	1	*0.0040*
Ordovician–Silurian	0.0617	1	0.7649	0.9806
Silurian–Devonian	1	0.7291	1	0.6568
Devonian–Carboniferous	0.2621	0.1966	0.2286	0.0615
Carboniferous–Permian	1	*0.0019*	*0.0325*	0.2682
long-term trends				
Cambrian–Ordovician	*0.0026*	0.7291	1	*0.0040*
Cambrian–Silurian	0.9979	0.7291	0.7649	*0.0409*
Cambrian–Devonian	0.9979	*0.0251*	0.7649	*0.0006*
Cambrian–Carboniferous	0.0617	*0.0001*	*0.0047*	*0.0000*
Cambrian–Permian	0.0617	*0.0000*	*0.0000*	*0.0000*
Ordovician–Silurian	0.0617	1	0.7649	0.9806
Ordovician–Devonian	0.0617	0.7291	0.7649	0.9999
Ordovician–Carboniferous	0.9979	*0.0019*	*0.0047*	*0.0114*
Ordovician–Permian	0.9979	*0.0000*	*0.0000*	*0.0000*
Silurian–Devonian	1	0.7291	1	0.6568
Silurian–Carboniferous	0.2621	*0.0019*	0.2286	*0.0013*
Silurian–Permian	0.2621	*0.0000*	*0.0003*	*0.0000*
Devonian–Carboniferous	0.2621	0.1966	0.2286	0.0615
Devonian–Permian	0.2621	*0.0001*	*0.0003*	*0.0002*

In order to determine if the median number of segments changed significantly through time, we applied the Richter & McCann [16] single-step approach to segment counts in the thorax, pygidium and trunk, as well as the proportion of segments in the trunk allocated to the pygidium, across two timescales (periods and series/subsystems). In order to determine if detected shifts in segment counts were associated with taxonomic turnover, we repeated the analysis in a jackknife fashion (removing one group at a time). We also applied the single-step approach to two taxonomic groups, the Phacopida and Proetida (sensu [19]), which have relatively stable taxonomic status and large sample sizes. In order to determine if the mode of enrolment was associated with changes in segment counts, we subset the data for taxa where enrolment type is known (electronic supplementary material, Dataset S1) and applied the single-step approach to segment counts in the thorax and the proportion of segments allocated to the pygidium, within each mode across geological periods. The size of the subset of data for which enrolment type was available was too small to allow for testing at the higher resolution timescale and within some enrolment types.

All data analysis and visualization were done in the statistical programming language R v4.2.2 [20] using the R packages ggplot2 v3.4.0 [21], ggpubr v0.4.0 [22], moments v0.14.1 [23], rapportools v1.1 [24], scripts from [25] and original scripts (electronic supplementary material, Dataset S6).

## 3. Results and discussion

**Figure 2.**
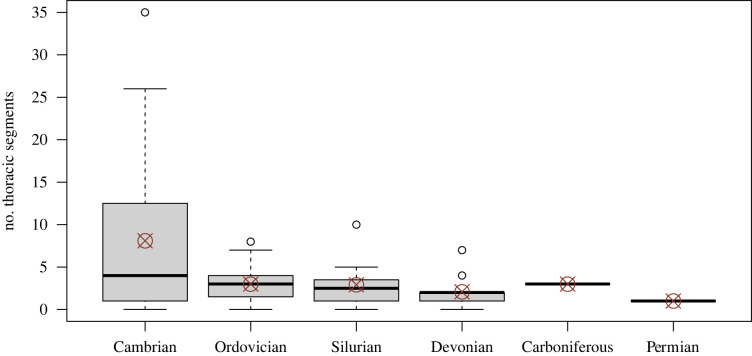
Box plots showing a decrease through time in the within-family range in the number of segments in the thorax. The median (thick black horizontal bar) and mean (red circle with superimposed X) range decreases through time. Thus within families, the number of segments in the thorax becomes increasingly more constrained and therefore more conservative throughout the Palaeozoic. The pattern occurs despite an increase in the number of species sampled within families (electronic supplementary material, figure S8). (Online version in colour.)

Applying a permutation test for multiple groups [16,26] to a dataset of segment counts for 1589 trilobite species, we found that the median number of segments in the thorax decreased significantly from the Cambrian to the Ordovician, then increased marginally significantly from the Ordovician to the Silurian (figure 1*a*, table 1; electronic supplementary material, table S4). The decrease from the Cambrian to the Ordovician appears to be the result of a protracted decrease across the Cambrian and into the Middle Ordovician (see results for finer timescale; electronic supplementary material, figure S6 and table S6). Perhaps more notable is the contraction of the range in the number of segments in the thorax, which is brought about by both a decrease in the maximum number and increase in the minimum number over the Palaeozoic (figure 1*a*). Subsampling indicates that the contraction in range is not an artefact of differences in sample size across time intervals (electronic supplementary material, figure S7). This contraction is consistent with an increase in taxonomic conservation in the number of segments in the thorax within subclades (figure 2; see also electronic supplementary material, figure S8) [27,28].

The median number of segments in the pygidium increased steadily across the Palaeozoic (figure 1*b*; electronic supplementary material, figure S6), with the largest and most significant increases occurring in the late Palaeozoic (table 1; electronic supplementary material, table S6). Both the minimum and maximum numbers increased, but overall the range was relatively constant, with the exception of a large spike in the maximum during the Silurian related to the inclusion of encrinurids with large numbers of axial rings (see electronic supplementary material).

As a result of the above changes, the median number of segments in the trunk remained essentially constant until the late Palaeozoic but the range in segment number decreased considerably (figure 1*c*; electronic supplementary material, figure S6 and table S6; table 1). The proportion of segments in the pygidium relative to the thorax increased significantly from the Cambrian to the Ordovician and again (though less significantly) from the Devonian to the Carboniferous (figure 1*d*, table 1; electronic supplementary material, figure S6 and table S6). There was also less variation in the proportion of the trunk comprised by the pygidium over time (figure 1*d*).

**Figure 3.**
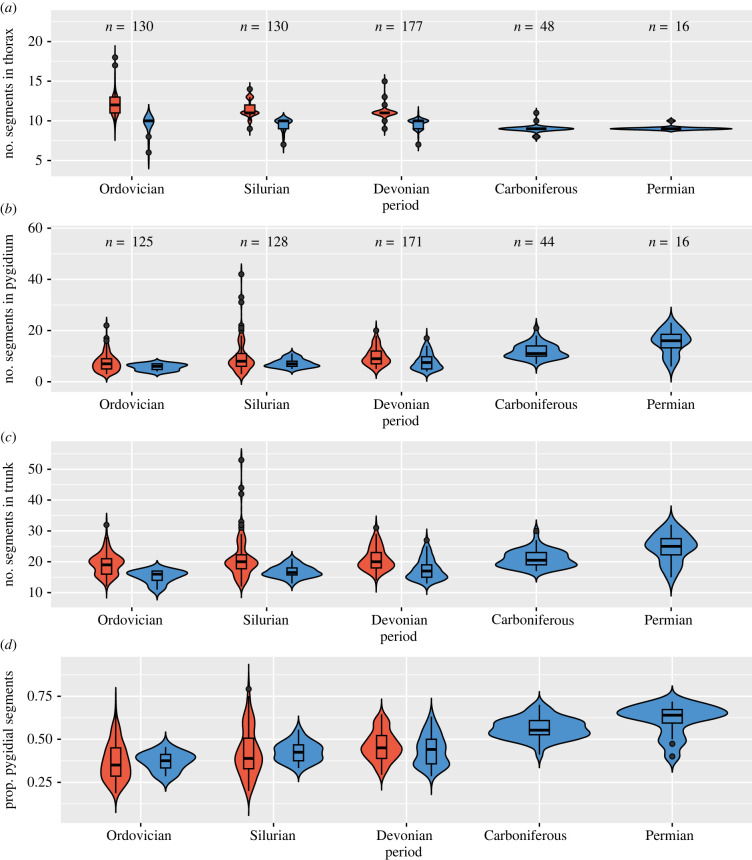
Violin and box plots showing the distribution and median/quartiles, respectively, in segment count in the thorax (*a*), pygidium (*b*), entire trunk (*c*) and proportion of the trunk segments that are fused in the pygidium (*d*) within and order Phacopida (left-hand plots in red for Ordovician to Devonian) and order Proetida (*sensu* Adrain [19], which includes the families Proetidae and Tropidocoryphidae; right-hand plots in blue for Ordovician to Permian). Sample sizes for each period are shown at top of each panel; sample sizes in (*c*,*d*) are the same as (*b*).

The lack of taxonomic consensus among trilobite systematists and a paucity of robust clade-wide trees ([12,29,30], see also electronic supplementary material) precludes extensive investigation of patterns within a phylogenetic framework. However, a jackknife procedure revealed that taxonomic turnover of both older and younger clades had specific impacts on shifts in the median number of segments in the thorax and pygidium (electronic supplementary material, tables S12 and S13). For example, the significant decrease from the Cambrian and modest increase from the Ordovician in the median number of segments in the thorax appears to be driven by the appearance and disappearance of asaphides (sensu [19], which includes trinucleides), which comprise 46% of the Ordovician sample and are dominated by species with small numbers of thoracic segments (median = 8 for asaphides, 6 for trinucleides). As would be expected given their dominance in the late Palaeozoic, the exclusion of the Proetida (sensu [19]), which includes the ‘phillipsiids’ of the mid-Carboniferous and Permian [31], eliminates all significant changes in the median number of segments in the thorax and pygidium. However, the late Palaeozoic increase is not due only to the reduction of the clade during the late Devonian extinctions: the proetids show a long-term increase in median number of pygidial segments and proportion of trunk segments comprised by the pygidium that predated the extinction of other trilobite subclades (figure 3). Similarly, shifts in segment number and identity seen in the order Phacopida, a diverse and long-lived subclade whose status as a monophyletic group is uncontroversial (see electronic supplementary material), are consistent with patterns documented across the clade (figure 3). Although small sample size precludes the statistical testing for differences in medians at lower taxonomic levels, there is some evidence for a variety of different patterns. For example, within the family Dalmanitidae, the median and range in the number of thoracic segments stays the same from the Ordovician to the Devonian, but the number of pygidial segments noticeably increases from the Silurian to the Devonian (electronic supplementary material, figure S9). By contrast, the median and range decrease in both the thorax and pygidium within the family Ceratopygidae between the Cambrian and Ordovician (electronic supplementary material, figure S9). Within the family Styginidae, the median number of segments in both the thorax and pygidium increases from the Ordovician to the Silurian; by the Devonian, the number in the thorax is constant but there is still minor variation in the number of pygidial segments (electronic supplementary material, figure S9). By contrast, the range in number of pygidial segments in Proetidae increases dramatically in the late Palaeozoic (electronic supplementary material, figure S9). Finally, independent studies at the genus and family level indicate that the number of thoracic and/or pygidial segments can shift within lineages [28,32]. Based on these observations, it seems unlikely that shifts in segment number and allocation in the body are simply passive realizations of taxonomic turnover.

**Figure 4.**
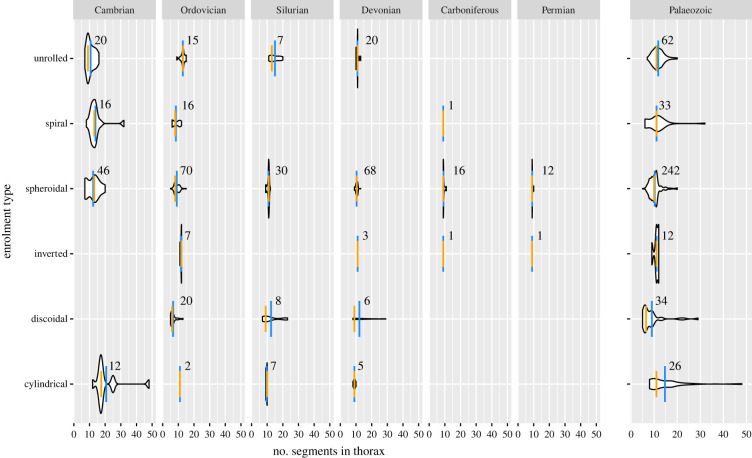
Number of segments in the thorax for trilobites with different enrolment strategies [39]. Short orange line = median; long blue line = mean; numbers adjacent to distributions represent sample sizes. See electronic supplementary material, figure S10 for proportions of pygidial segments and electronic supplementary material, tables S14–S16 for results of statistical analysis.

A candidate ecological explanation lies in long-term changes that occurred in enrolment behaviour in trilobites. Enrolment is a posture seen in trilobite specimens whereby the pygidium and cephalon were brought in close proximity. Frequently, the cephalon and pygidium are in complete alignment and the exoskeleton would have completely enclosed the ventral side of the body including appendages. By analogy to modern arthropods, like terrestrial isopods, enrolment is interpreted to have been a defensive behaviour. Evidence of the long-term adaptive value of this behaviour comes from the appearance of features on the margins of the cephalon and pygidium, such as grooves, that facilitated a tight fit devoid of gaps [33–37]. Several enrolment types have been observed across trilobites. The greatest variety of types occurred in the Cambrian, and there was a decrease in the number of enrolment strategies as well as an increase in the dominance of some strategies over the Palaeozoic [38,39]. In addition to local coadaptive features on sclerite margins, shifts in enrolment type may have been facilitated by changes in segment number and allocation in the body, which would have impacted the flexibility of the trunk region as well as contributed to ‘caudalization’ [40,41]. We used the dataset of Suárez & Esteve [39] to investigate changes in enrolment strategies as a possible driver for the contraction in thoracic segment range and increased proportion of the trunk segments being allocated to the pygidium. Across the Palaeozoic, there is no significant difference in the median number of segments in the thorax with the exception of the discoidal strategy, which had a significantly lower median number of tergites compared to species using other enrolment strategies (figure 4; electronic supplementary material, table S14). This difference appears to be largely driven by the inclusion of Ordovician trinucleide families with low thoracic tergite counts in this group (47%). The species with spheroidal enrolment had the largest proportion of the trunk segments allocated to the pygidium, but only significantly so compared to species with the cylindrical mode (electronic supplementary material, figure S10 and table S14). The median and range in the proportion of segments allocated to the pygidium was similar among Cambrian species regardless of enrolment strategy, and although there was some increased separation among strategies, medians and ranges were similar for the most common types (electronic supplementary material, figure S10). Thus, although the spheroidal strategy increased in prevalence across the Palaeozoic, taxa showing this enrolment strategy were not apparently limited to having a smaller number of segments in the thorax and/or larger proportion of segments allocated to the pygidium. Furthermore, trends seen across the clade are mirrored within subgroups defined by enrolment strategy. For example, there was a significant decrease in the number of segments in the thorax and a significant increase in the proportion of segments allocated to the pygidium between the Cambrian and Ordovician within groups defined by enrolment type (figure 4; electronic supplementary material, tables S15 and S16), consistent with these changes having occurred across trilobites regardless of enrolment strategy. These results suggest that change in the prevalence of certain enrolment strategies does not provide strong explanatory power for associated shifts in segment number and allocation.

In 1839, Hermanus Emmrich observed that trilobites with large numbers of thoracic tergites often had small numbers of pygidial segments, and vice versa, and proposed a ‘law’ of a constant 21 segments in the trilobite body ([42], original in Latin, paraphrased in [8]). Although this idea was ‘brought…into total disrepute’ [8, p. 128] by the accumulation of numerous exceptions, a more probabilistic version of Emmrich's observations is supported by the analysis herein: on average, the number of segments in the trilobite body did not change until the latest Palaeozoic. In fact, we find a median (and mean) of 17 segments across the entire clade and a long-term convergence towards the median over time. Within this constraint, there was a long-term shift in segment identity as an increasing proportion of generated segments remained fused within the pygidium over ontogeny. Assessment at multiple taxonomic levels indicates that these trends are not due simply to taxonomic turnover resulting from unrelated or random processes. Although segmentation patterns in the trilobite body may have so far unidentified ecological importance, including the possible ecological importance of the increased ‘distinctiveness’ of the pygidium [11], previous hypotheses about the role of enrolment strategy in driving these trends remain unsubstantiated.

## Data Availability

All datasets and scripts supporting this study are available from the Dryad Digital Repository: https://doi.org/10.5061/dryad.rv15dv4bf [43]. Supplementary text, figures and tables are provided as electronic supplementary material [44].

## Endnote

1 Detailed description of the ventral exoskeleton and appendage attachment sites in conjunction with comparative developmental studies support segmental mismatch and the indirect expression of segmental patterns in the thorax. Although trilobite palaeontologists have historically referred to each of the biomineralized dorsal sclerites in the thorax as a ‘thoracic segment’, these sclerites would be more appropriately referred to as thoracic ‘tergites’. Nonetheless, different proposed correspondences between dorsal sclerites, ventral sclerites, and developmental segments do not lead to different segment counts in the thorax, so in terms of segment number, ‘thoracic tergite’ and ‘thoracic segment’ may be used interchangeably.
